# Physiological cell bioprinting density in human bone-derived cell-laden scaffolds enhances matrix mineralization rate and stiffness under dynamic loading

**DOI:** 10.3389/fbioe.2024.1310289

**Published:** 2024-02-14

**Authors:** Anke M. de Leeuw, Reto Graf, Pei Jin Lim, Jianhua Zhang, Gian Nutal Schädli, Sheila Peterhans, Marianne Rohrbach, Cecilia Giunta, Matthias Rüger, Marina Rubert, Ralph Müller

**Affiliations:** ^1^ Institute for Biomechanics, ETH Zurich, Zurich, Switzerland; ^2^ Connective Tissue Unit, Division of Metabolism and Children’s Research Center, University Children’s Hospital Zurich, University of Zurich, Zurich, Switzerland; ^3^ Department of Pediatric Orthopaedics and Traumatology, University Children’s Hospital Zurich, Zurich, Switzerland

**Keywords:** cell-laden scaffold, cell density, 3D bioprinting, dynamic culture, time-lapsed micro-CT

## Abstract

Human organotypic bone models are an emerging technology that replicate bone physiology and mechanobiology for comprehensive *in vitro* experimentation over prolonged periods of time. Recently, we introduced a mineralized bone model based on 3D bioprinted cell-laden alginate-gelatin-graphene oxide hydrogels cultured under dynamic loading using commercially available human mesenchymal stem cells. In the present study, we created cell-laden scaffolds from primary human osteoblasts isolated from surgical waste material and investigated the effects of a previously reported optimal cell printing density (5 × 10^6^ cells/mL bioink) vs. a higher physiological cell density (10 × 10^6^ cells/mL bioink). We studied mineral formation, scaffold stiffness, and cell morphology over a 10-week period to determine culture conditions for primary human bone cells in this microenvironment. For analysis, the human bone-derived cell-laden scaffolds underwent multiscale assessment at specific timepoints. High cell viability was observed in both groups after bioprinting (>90%) and after 2 weeks of daily mechanical loading (>85%). Bioprinting at a higher cell density resulted in faster mineral formation rates, higher mineral densities and remarkably a 10-fold increase in stiffness compared to a modest 2-fold increase in the lower printing density group. In addition, physiological cell bioprinting densities positively impacted cell spreading and formation of dendritic interconnections. We conclude that our methodology of processing patient-specific human bone cells, subsequent biofabrication and dynamic culturing reliably affords mineralized cell-laden scaffolds. In the future, *in vitro* systems based on patient-derived cells could be applied to study the individual phenotype of bone disorders such as osteogenesis imperfecta and aid clinical decision making.

## 1 Introduction

Bone constitutes a dynamic composite material with a hierarchical macro- and microstructure. The extracellular matrix of bone mainly comprises type 1 collagen fibers that provide tensile strength and are reinforced with hydroxyapatite mineral to add compressive strength. Osteoblasts secrete type I collagen and other matrix organizing proteins to form osteoid which is subsequently mineralized. Osteoclasts resorb bone by dissolving minerals and enzymatically digesting the matrix. The process of bone remodeling is tightly regulated by mechanosensing osteocytes, which form an interconnected network and signal to osteoblasts and osteoclasts to generate or degrade bone in response to mechanical stimuli (Allen, 2014). *In vivo*, bone tissue is subjected to complex static and dynamic loads that result in mechanical strain and fluid shear stresses within the canalicular network of osteocytes. In metabolic bone disease bone homeostasis is disturbed, which can result in fractures, deformities or arthropathy (Bartl, 2017). With current diagnostic technology, alterations in bone biology and tissue biomechanics cannot be reliably captured, making precise predictions of individual disease trajectories difficult. Considering the profound impact of musculoskeletal disorders on health systems at large, advanced *in vitro* models for delineating patient-specific pathomechanisms and developing personalized therapies are currently lacking (McConaghy et al., 2023). Such advanced *in vitro* bone models, that more closely mimic the human bone microenvironment, may offer clinically relevant platforms for rare bone diseases such as osteogenesis imperfecta.

Comprehensive multicellular *in vitro* models appear to be a promising technology for overcoming these predicaments. Organotypic models are defined as 3D tissue constructs resembling the *in vivo* condition, that enhance our understanding of the development, growth, and function of organs (Shamir and Ewald, 2014; Malik and Mukherjee, 2022). While organotypic bone technology is still in its infancy, significant advances were made in recent years (Owen and Reilly, 2018; Iordachescu et al., 2019; Yuste et al., 2021; Huang et al., 2023). Constructs vary in cell source, scaffold material, construct size, fabrication technique and culture conditions. As any living multicellular system operates in three-dimensional space, 3D *in vitro* models improve upon conventional 2D cell culture by providing a microenvironment and architecture that supports physiological cell functionality and self-organization. Mimicking the complex properties of the extracellular matrix is of critical importance. This entails parameters such as collagen composition, topology, crosslinking, and stiffness, as well as enzymatic degradability. Consequently, *in vitro* bone models require suitable 3D scaffolds laden with primary bone cells from either commercial sources or patient donors (Amini et al., 2012) to create a microenvironment amenable to mechanical loading and mineralization. Scaffolds for culturing human donor cells have been fabricated by salt leaching (Akiva et al., 2021), extrusion bioprinting (Zhang et al., 2022), sintering (Bourgine et al., 2018) or using decellularized bone matrices (Iordachescu et al., 2021).

Bone requires weeks to months, oftentimes years, under physiological loading to mature and develop its mechanical properties and unique architecture. One major advantage of *in vitro* bone model systems is the extended culture periods to enable higher degrees of maturation and mineralization. Mechanical stimulation as one of the key drivers for bone development *in vivo* needs to be incorporated in *in vitro* systems to enable maturation of the construct that include changes in cell biology and structural morphology over time. As bone physiology *in vivo* is highly dependent on mechanical cues, comprehensive *in vitro* bone models include bioreactor systems that simulate mechanical loading at physiological or even supraphysiological levels (Owen and Reilly, 2018; Scheinpflug et al., 2018) to trigger cell differentiation and proliferation and drive specific activities such as matrix deposition and mineralization (Zhang et al., 2021b). Technically, mechanical loading is implemented by spinner, perfusion, compression or rotational (NASA Synthecon) bioreactors exerting fluid shear stress, cyclic compressive loading, or microgravity forces, respectively (Bourgine et al., 2018; Akiva et al., 2021; Iordachescu et al., 2021; Schadli et al., 2021; Mainardi et al., 2022). Bioreactor systems have been utilized to improve the osteogenic development and mineralization of *in vitro* bone models by subjecting human mesenchymal stem cells (hMSCs) to mechanical stimuli such as fluid shear stress or compressive loading (Akiva et al., 2021; Zhang et al., 2022). Fluid shear stress of the interstitial fluid in the lacunar-canalicular network of bone is thought to stimulate mechanoreceptors on osteocytes to signal osteoblasts to start bone formation (Wittkowske et al., 2016; Qin et al., 2020). Similarly, compressive loading is thought to induce fluid flow and cause deformation of the osteocyte cytoskeleton, triggering an intracellular signaling pathway that decreases sclerostin production and upregulates osteoblast activity (Qin et al., 2020).

**FIGURE 1:**
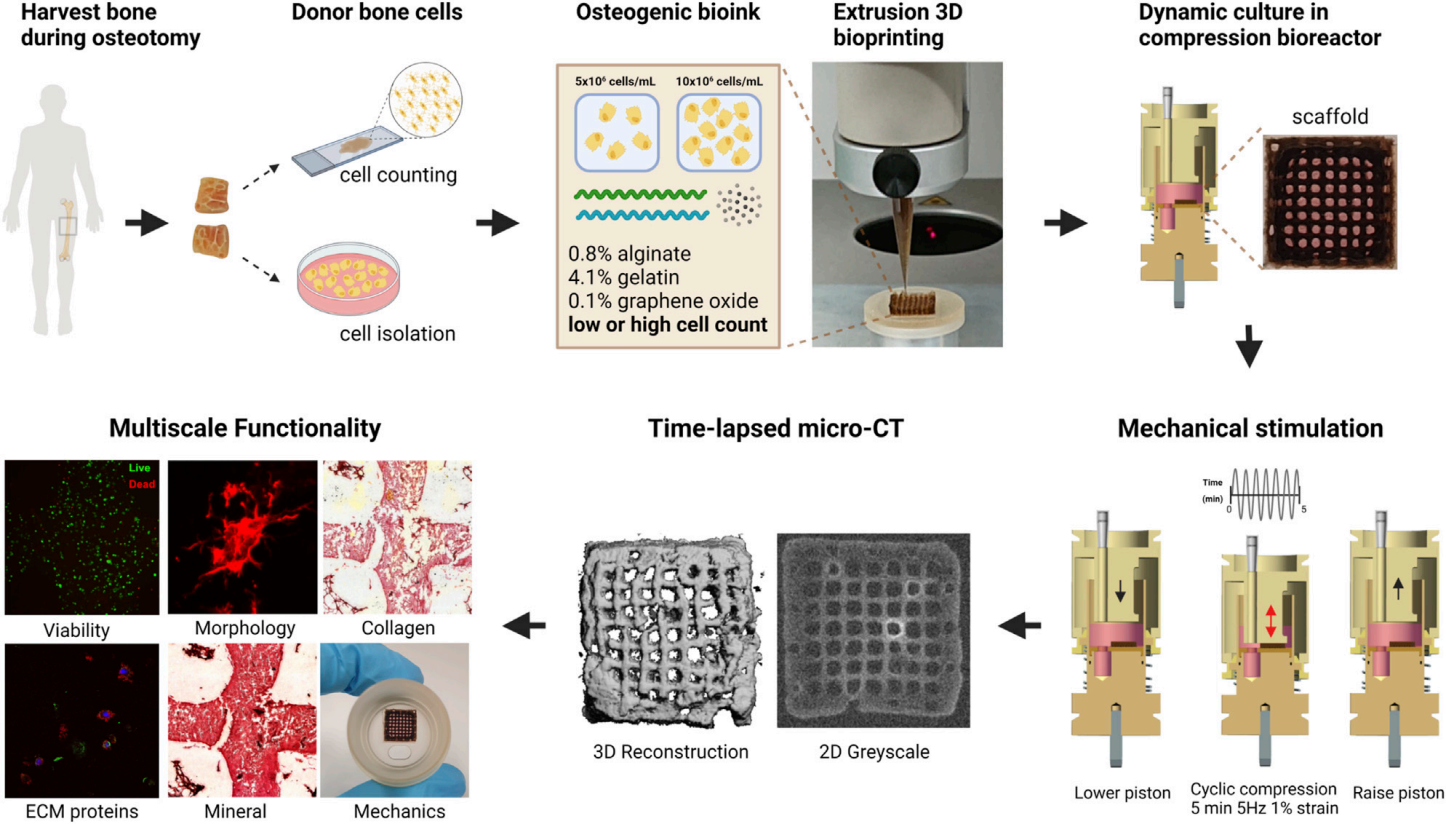
Schematic of cell-laden scaffold pipeline. Bone is collected as waste material during orthopedic surgery, then segmented and prepared for primary osteoblast isolation or fixation to establish the donor’s cell density. Donor osteoblasts are encapsulated in an osteogenic bioink containing 0.8% alginate 4.1% gelatin 0.1% graphene oxide microparticles. Cells were extruded at 5 × 10^6^ (low cell density) or 10 × 10^6^ (high cell density) cells/mL of hydrogel and cultured in compression bioreactors for up to 10 weeks. Integration of time-lapsed micro-CT scans alongside traditional assays facilitates the multiscale assessment of cell-laden scaffold functionality. Figure created using BioRender.com.

The main hurdle of *in vitro* bone models is to produce stable dynamic systems that accurately replicate *in vivo* conditions and provide reliable biomarkers for correlation with clinical phenotypes and disease trajectories. As initial cell printing density influences mineralization and cell-cell interactions (Zhang et al., 2020) as well as osteogenic development and maturation of *in vitro* bone models (Zhou et al., 2011; Maia et al., 2014; Yassin et al., 2015), it is critical to investigate its effect when establishing a personalized organotypic bone model. In a preceding study, our group established an *in vitro* bone model using commercially available hMSCs cultured under dynamic compressive loading in a purpose-built bioreactor system to produce 3D functional osteocyte bone organoids (Zhang et al., 2022). In this microenvironment, constructs demonstrated robust cell differentiation and mineralization over an 8-week period. The clinical translatability of this approach using hMSCs as the primary cell source is constrained by the lack of sufficient number of hMSCs available from bone biopsies or tissue samples. To develop a personalized model, we need a patient-derived cell source. Harvesting hMSCs for the sole purpose of this study by means of a dedicated surgical intervention would be ethically prohibitive (Hernigou et al., 2014). It is therefore imperative to develop a patient cell isolation process that integrates into existing clinical pathways without the need for an additional surgery for cell harvesting. To address this gap between technology and clinical practice, and advance *in vitro* bone models closer to becoming a clinically translatable model, we introduce a methodology employing primary bone cells sourced from surgical waste material as part of already planned surgeries. Once validated, the model and associated methods will be applied to pediatric populations with skeletal dysplasias and other chronic bone disorders including osteogenesis imperfecta. In the present study, we investigated if (i) the 3D bioprinting pipeline negatively affects primary human bone cells obtained directly from patients, and (ii) initial cell printing density affects subsequent cell viability, morphology, osteogenic protein expression, overall mineral formation, and stiffness over an extended, 10-week period. An overview of the entire pipeline is given in Figure 1.

## 2 Materials and methods

### 2.1 Origin of primary cells and cell lines

This study was conducted according to the Declaration of Helsinki for Human Rights, in the presence of a signed informed consent of the patient or his parents for the use of biological material for research studies. Approval from Swiss Ethics (Kantonale Ethikkommission Zürich, KEK Nr. 2014–0300 and Nr. 2019–00811) has been granted to CG and MRo for biochemical and molecular studies on patients’ biological material. Firstly, an entire thickness segment was collected from a femur osteotomy of a healthy 15-year-old male donor with limb malalignment as waste material under the study protocol approved by Swiss Ethics. Next, the biosample was prepared for cell isolation or fixation. The fixed bone segment represents the reference and positive control, while the isolated primary osteoblasts serve as the starting material for cell-laden scaffolds.

### 2.2 Establishing primary osteoblast cultures from bone explants

**FIGURE 2:**
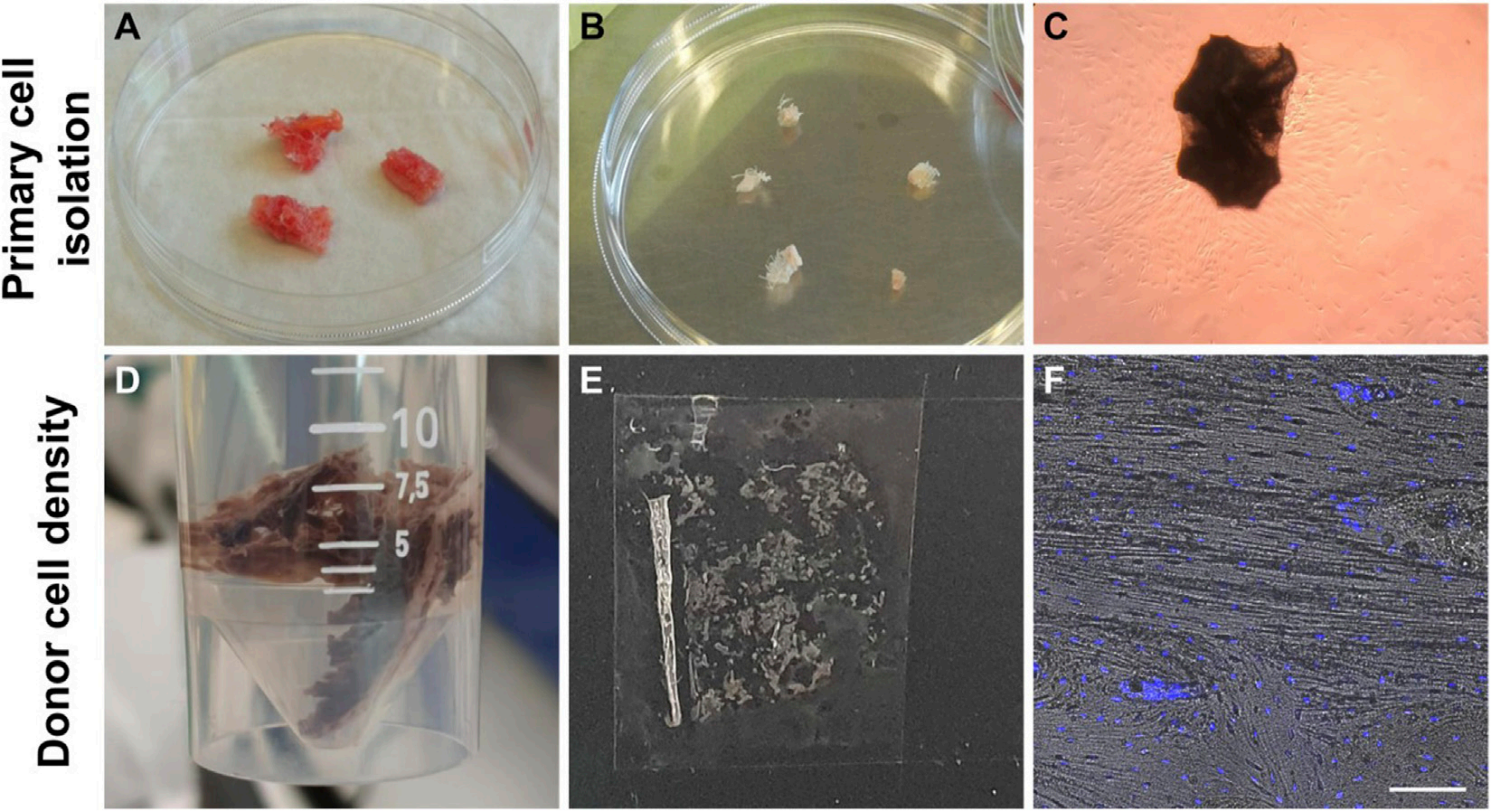
Workflow of establishing primary osteoblasts from bone explants **(A–C)**. Workflow of reference bone preparation to establish donor’s bone cell density **(D–F)**. **(A)** Bone explants after a single PBS wash. **(B)** Bone explants after cutting into 10—20 mm long pieces and washing in PBS five times. **(C)** Cells that migrated out of the bone explants and attached to the culture dishes were observed after 1 week in culture. **(D)** Fixation of bone segment in 4% PFA. **(E)** Cryosection of human bone. **(F)** Hoechst-stained cell nuclei and brightfield image overlay used to count cells and establish the average cell density of this donor. Scale bar = 50 µm.

The bone explants were transferred from the surgery room to the laboratory in a sealed tube containing Dulbecco’s Modified Eagle Medium (DMEM, Gibco) at room temperature and processed on the same day for establishing osteoblast cultures or fixed for histological staining as summarized in Figure 2. The bone explants were rinsed in 10 mL Phosphate Buffered Saline (PBS, Gibco), cut into approximately 10–20 mm long pieces, and transferred to a 50 mL Falcon tube containing 10 mL fresh PBS. The samples were vortexed thrice for 10–15 s each, allowed to stand for 30 s and the PBS was removed by aspiration after that. This washing step was repeated five times until most of the blood contaminants were removed. Subsequently, the bone explants were transferred to sterile 10 cm tissue culture dishes (Sarstedt) and cultured at 37°C and 5% CO_2_ in DMEM supplemented with 10% fetal bovine serum (FBS, Gibco), and antibiotic-antimycotic (Gibco, containing 100 U/mL penicillin, 100 mg/mL streptomycin, and 0.25 mg/mL Amphotericin B). The explants were left undisturbed for 7 days, and the culture medium was changed every 3–4 days thereafter. Cells that migrated out of explants and attached to the culture dishes were dislodged by trypsinization and expanded in T75 culture flasks until they reached 90% confluency, after which they were cryopreserved in FBS and 10% dimethyl sulfoxide (DMSO) until further analyses.

### 2.3 Establishing donor’s physiological cell density

Following surgery, a 2.5 cm × 1.5 cm x 2 cm bone explant sample was washed in PBS and fixed in ice-cold 4% paraformaldehyde (PFA) for 48 h, followed by three PBS washes (Figures 2D–F). Then, the sample was decalcified in 12.5% EDTA (pH 7.4–7.6) for 2 weeks at 4°C (decalcification confirmed by scout view in microCT40 (SCANCO Medical AG, Brüttisellen, Switzerland)). Next, the decalcified bone was washed three times in PBS and dehydrated in ice-cold 20% sucrose and 2% Polyvinylpyrrolidone (PVP) for 3 days. The sample was further dehydrated in 30% sucrose and 3% PVP solution for 3 weeks. Finally, the bone was embedded in optimal cutting temperature compound (OCT, VWR) and flash frozen in liquid nitrogen. The bone was cryosectioned (10–50 µm thickness) using Kawamoto’s cryofilm type 2C (SECTION-LAB Co. Ltd., Japan) using a cryotome (CryoStar NX70, Thermo Scientific) (Dyment et al., 2016). Then sections were adhered to microscope slides (SuperFrost™ Microscope Slides, ThermoScientific) using 1% (w/v) chitosan in 1% (v/v) acetic acid. Cryosections (50 μm) were washed three times in PBS for 5 min and permeabilized in 0.1% Triton-X-100 in PBS for 15 min. Sections were washed three times in PBS and incubated with 1:1000 Hoechst 33342 (1:200, B2261, Sigma-Aldrich) in 0.1% BSA in PBS for 1h. Sections were washed again three times in PBS and mounted with Fluoroshield for confocal imaging (Leica SP8). Osteocyte density (cells/mm^2^) was determined from two different locations in stained cryosections (*n* = 6) using the Cell Counter Fiji Plugin and verified by manual counting (Figure 2F). The mean cell density of osteocytes in the donor bone benchmark was 359 ± 74.6 cells/mm^2^ (Supplementary Table S1).

### 2.4 Osteoblast expansion

One cryovial containing 5 million cells (Passage 5) was rapidly thawed in a 37°C water bath. Cells were transferred to a 50 mL Falcon tube containing 30 mL of DMEM and centrifuged at 300 *g* for 10 min at 4°C. The cell pellet was resuspended in 40 mL of expansion medium, and 10 mL was added to each triple flask containing 90 mL of expansion medium (DMEM, 10% FBS, 1% antibiotic-antimycotic, 1% non-essential amino acid and 1 ng/mL basic fibroblast growth factor) under standard culture conditions (37°C, 5% CO_2_) for 7 days prior to bioprinting.

### 2.5 Bioink preparation and 3D bioprinting

Passage 5 primary osteoblasts were harvested by incubation with 0.25% Trypsin-EDTA and resuspended in the control medium (DMEM, 10% FBS, 1% Anti-Anti). Cell suspensions were kept on ice and, when needed, centrifuged, and resuspended in 60uL of control medium. Bioinks with low cell density (5 million cells/mL, serving as control group) and high cell density (10 million cells/mL, representing the more physiologically relevant group) were prepared by mixing cell suspensions with 1 mL 4.1% (w/v) gelatin, 0.8% (w/v) alginate and 0.1% (w/v) graphene oxide hydrogels as described previously (Zhang et al., 2021a). A control group of 5 × 10^6^ cells/mL bioink was included based on previous work (Zhang et al., 2020), where the initial mineral formation rate was increased in 5 × 10^6^ cells/mL hMSC-laden scaffolds compared to other cell densities (0, 1.67 × 10^6^ and 15 × 10^6^ cells/mL scaffolds). Bioinks were loaded into 3-mL polyethylene cartridges fitted with 27-gauge tapered tips (Nordson EFD, Vilters, Switzerland). The 10 mm × 10 mm × 2.4 mm scaffolds were printed using a 3DDiscovery bioprinter (RegenHU; Villaz-St-Pierre, Switzerland) with a pneumatic dispenser onto double-sided tape (3M, Scotch, United States of America) taped on the bioreactor platform, as previously described (Zhang et al., 2022). The bioprinted structure consists of macroscale filaments (extruded hydrogel material forming a lattice) with cells embedded inside the hydrogel and pores (empty spaces between printed filaments). Scaffolds on platforms were crosslinked with 2% (w/v) calcium chloride in the control medium for 10 min, then washed twice in the control medium. Scaffolds were transferred to 6-well plates with fresh control medium and incubated at 37°C and 5% CO_2_.

### 2.6 Compression bioreactor culture

The day after bioprinting, scaffolds were assembled into custom-made polyetherimide compression bioreactors. Each bioreactor was filled with 5 mL osteogenic medium (DMEM, 10% FBS, 1% Anti-Anti, 50 μg/mL ascorbic acid, 100 nM dexamethasone, 10 mM β-glycerophosphate) with media changes performed three times per week. Scaffolds were individually cyclically loaded in a mechanical stimulation unit (MSU) controlled via an in-house program on LabView (National Instruments, Austin, Texas). The loading protocol consisted of uniaxial compression loading with a preload of 0.07 N and a sinusoidal strain amplitude of 1% at a frequency of 5 Hz for 5 min 5 times per week (Zhang et al., 2022). Cell-laden scaffolds were cultured for up to 10 weeks.

### 2.7 Cell viability

LIVE/DEAD^®^ Viability/Cytotoxicity assay was tested on scaffolds (*n* ≥ 3) after 1 day of bioprinting and after 15 days of compression loading to assess the impact of bioprinting and compression loading on cell viability after bioprinting and after 2 weeks of compression loading. Briefly, scaffolds were incubated with 2 μM Calcein AM and 4 μM ethidium homodimer for 40 min 37°C and 5% CO_2_. Then, scaffolds were washed twice with pre-warmed PBS and transferred to 8-well chamber slides (Ibidi GmbH, Germany) for imaging using a confocal microscope (Visitron Spinning Disc, Nikon Eclipse T1). For each scaffold, 6 distinct regions were imaged. Cell viability was calculated using ImageJ (National Institutes of Health, United States of America) as the ratio of the number of living cells to the total number of cells. Cell density (cells/mm^2^) was estimated using ImageJ as number of living cells per scaffold area.

### 2.8 Time-lapsed micro-computed tomography

Bioreactors were scanned every 7 days in a micro-computed tomography (micro-CT) scanner (µCT45, SCANCO Medical AG, Brüttisellen, Switzerland) at a voxel resolution of 34.5 µm with an energy of 45 kVp, intensity of 177 μA, and an integration time of 600 m. The micro-CT voxels in grayscale images were converted to corresponding hydroxyapatite (HA) densities (mg HA/cm^3^) using the micro-CT manufacturer’s standardized calibration process. A mask was drawn around scaffolds to create a consistent volume of interest for the analysis. The same mask was used for all measurement days of the same scaffold. A constrained Gaussian filter (sigma 1.2, support 1) was applied using IPL Scanco AG software V5.42 to reduce image noise. We chose a global threshold of 97.5 mg HA/cm^3^ matching previous reports (Vetsch et al., 2017), to segment the mineralized ECM from the background (e.g., cell culture medium) visible by eye on the grayscale images at week 4. The mineral volume and density measurements from each timepoint were normalized by subtracting the first timepoint.

### 2.9 Scaffold mechanics

Scaffold mechanics were assessed using the in-house MSU as described previously (Schadli et al., 2021; Zhang et al., 2022). Daily non-destructive measurements, referred to as dynamic stiffness, were performed as part of the loading protocol to track scaffold mechanics over time. Unconfined uniaxial compression tests were performed under displacement control, with a preload of 0.07 N, and a displacement rate of 4 μm/s until the scaffold yielded. During compression the force and displacement were measured and fitted using Python (Python Software Foundation, Delaware, United States of America). From the fitted curve, the stiffness was calculated as the force per displacement at the steepest slope within the linear elastic region. Destructive measurements were performed on scaffolds (*n* = 3) at day 15, 30 and 70.

### 2.10 F-actin staining

F-actin cytoskeletal filaments were stained on day 30 and day 70 (endpoint) to assess cell spreading morphology. Briefly, scaffolds were removed from the incubator, washed twice in PBS, and fixed in 4% paraformaldehyde (PFA) in 10 mM calcium chloride and 0.15 M sodium chloride solution for 1 h. Samples were washed twice in PBS, blocked and permeabilized in 0.1% BSA 0.3% Triton-X-100 in PBS for 40 min. Samples were washed twice and incubated in Phalloidin-TRITC (1:100, P1951, Sigma-Aldrich) Hoechst (1:200, B2261, Sigma-Aldrich) in PBS for 50 min. Samples (*n* = 3) were washed twice in PBS and transferred to 8-well chamber slides (Ibidi GmbH, Germany) for imaging using a confocal microscope (Zeiss LSM 880 Airyscan, Germany). For each scaffold, 3 distinct regions were imaged and analyzed. Cell processes were manually measured using ZEN 2.3 (Carl Zeiss Microscopy software) to calculate the percentage of dendrites with length >10 µm as reported previously (Zhang et al., 2020). Actin fiber fluorescence area fraction was quantified from z-projections using ImageJ.

### 2.11 Scaffold sample preparation and histological staining

After 70 days of culture in compression bioreactors, scaffolds were rinsed twice with PBS and fixed with 4% PFA in 10 mM CaCl_2_ and 0.15 M NaCl solution for 2 h at room temperature. Samples were rinsed twice with 10 mM CaCl_2_ and 0.15 M NaCl solution and cryoprotected for 2 h with 10% sucrose in 10 mM CaCl_2_. Scaffolds were further cryoprotected in 30% sucrose in 10 mM CaCl_2_ overnight. Scaffolds were embedded in optimal cutting temperature compound (OCT, VWR) and flash-frozen in a methanol bath on dry ice. Samples were sectioned (10–30 µm thickness) using Kawamoto’s cryofilm type 2C (SECTION-LAB Co. Ltd., Japan) using a cryotome (CryoStar NX70, Thermo Scientific) (Dyment et al., 2016). Prior to staining, sections were adhered to microscope slides (SuperFrost™ Microscope Slides, ThermoScientific) using 1% (w/v) chitosan in 1% (v/v) acetic acid. Haematoxylin (Mayer’s, Sigma-Aldrich) and eosin (Y disodium salt, Sigma-Aldrich) (H&E) staining was performed to visualize cell nuclei, cytoplasm, and extracellular matrix. Alizarin Red S staining (2 mg/mL in acetone pH 4.3) (A5533, Sigma-Aldrich) was used to stain the mineralized extracellular matrix. Picrosirius red staining (365548, P6744, Sigma-Aldrich) enabled visualization of collagen. Histological sections were imaged with an automated slide scanner (Panoramic 250 Flash II, 3Dhistech, Hungary) at ×20 magnification.

### 2.12 Immunohistochemistry

Cryosections were washed three times in PBS for 5 min and permeabilized in 0.1% Triton-X-100 in PBS for 10 min. Sections were washed three times in PBS and blocked in 3% BSA in PBS for 1 h. Sections were incubated with primary antibody in 1% BSA in PBS overnight at 4°C. Antibody information is listed in Table 1. Sections were washed three times in PBS and incubated with secondary donkey anti-rabbit AF647 (1:1000, ab150075, Abcam) in 1% BSA in PBS for 1 h. F-actin was stained with Phalloidin in 1% BSA in PBS for 1 h, then sections were washed three times in PBS. Cell nuclei were stained with Hoechst 33342 (1:200, B2261, Sigma-Aldrich) in PBS for 15 min. Finally, sections were washed three times in PBS and mounted with Prolong Diamond Antifade Mountant (P36965, Invitrogen). Sections were sealed with nail polish and four to six distinct regions per sample were imaged using a confocal microscope (Zeiss LSM 880 Airyscan, Germany). For cell density (cells/mm^2^) assessment at day 70, Hoechst-stained nuclei were counted automatically using ImageJ in images of day 70 cryosections and verified by manual counting. Fluorescence area fraction (%) in immunostained cryosections was assessed using automatic Otsu thresholding in ImageJ.

**TABLE 1:** Information on antibodies and dyes used for immunohistochemistry.

Antibody/Dye	Dilution	Species	Supplier	Catalogue number
Osteocalcin	1:200	Rabbit	Abcam	ab93876
Sclerostin	1:200	Rabbit	Sigma-Aldrich	SAB1300753
Collagen I	1:200	Rabbit	Abcam	ab34710
Anti-Rabbit AF647	1:1000	Donkey	Abcam	ab150075
Phalloidin-TRITC	1:400	-	Sigma-Aldrich	P1951
Phalloidin AF555	1:500	-	Invitrogen	A34055
Hoechst 33342	1:200	-	Sigma-Aldrich	B2261

### 2.13 Scanning electron microscopy

Cryosections on Kawamoto’s tape were mounted on stubs (Plano GmbH, Germany) using conductive carbon adhesive stickers (Plano GmbH, Germany). Samples were sputter coated (CCU-010 Metal Sputter Coater Safematic GmbH, Switzerland) with a 5 nm Platinum/Palladium layer and imaged in a scanning electron microscope (Hitachi SU5000) using the secondary electron detector with an accelerating voltage of 5 kV.

### 2.14 Statistical analysis

Statistical analysis was performed using GraphPad Prism 9. * *p*-values less than 0.05 were considered statistically significant. Data are represented as mean ± standard deviation. Unpaired t-tests were performed to compare two groups. The difference in cell density was tested using a two-way ANOVA. The comparison of scaffold mineral density data at different timepoints was done using a two-way ANOVA followed by Sidak’s multiple comparisons test.

## 3 Results

### 3.1 Cell viability and density

**FIGURE 3:**
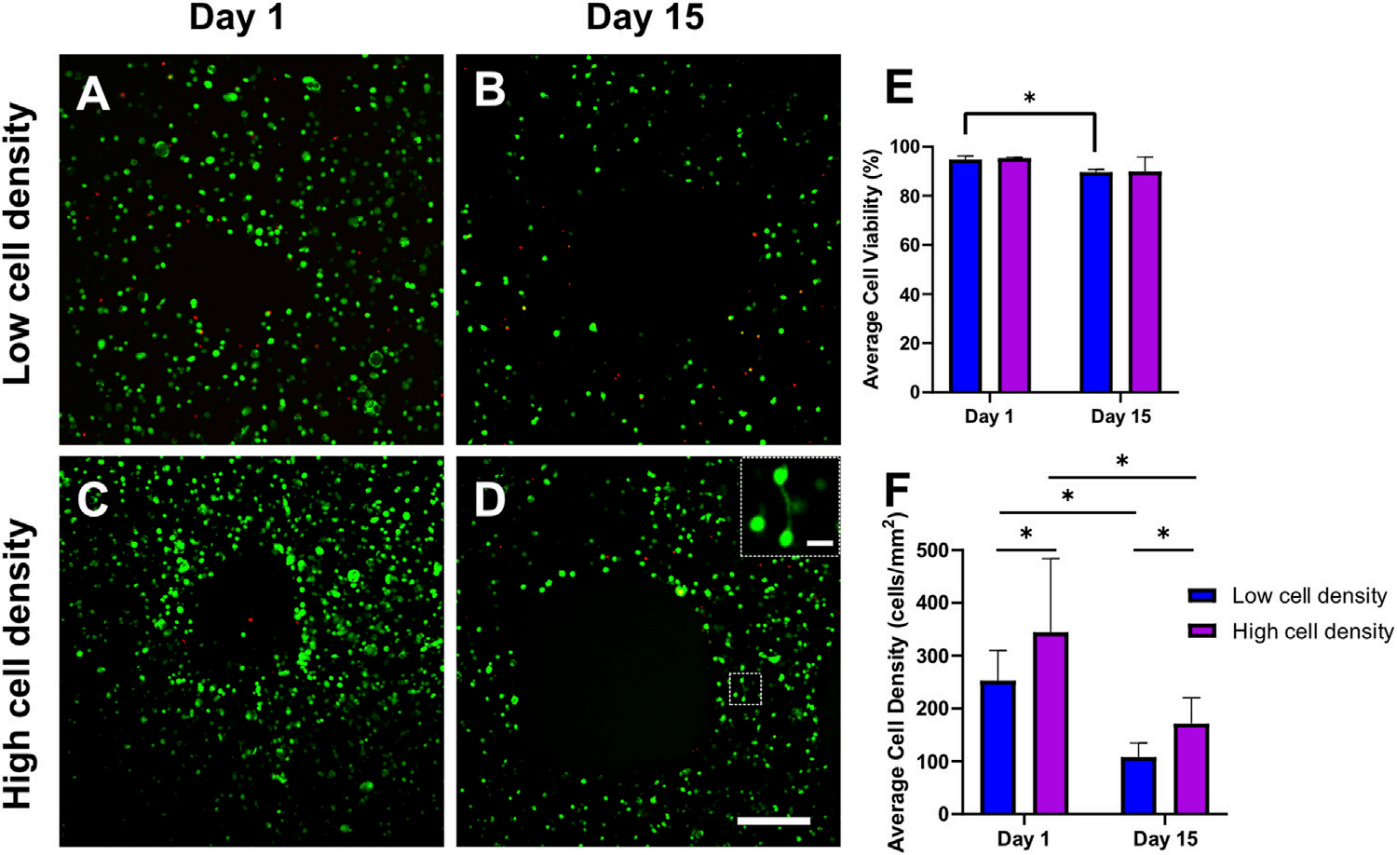
Representative fluorescent images of Calcein-AM/Ethidium homodimer-1-stained 3D bioprinted cell-laden scaffolds. Confocal images were taken after bioprinting (day 1) and after 2 weeks of daily mechanical loading (day 15) **(A–D)** to assess **(E)** average cell viability and cell density **(F)** in low and high cell density scaffolds. Scale bar = 250 µm. **(D)** High magnification insert indicates cell connection visible after 2 weeks of loading in high cell density scaffolds. Scale bar = 25 μm *p < 0.05, data are shown as the mean ± standard deviation (*n* = 3).

Figure 3 illustrates cell viability in Calcein-AM/Ethidium homodimer-1-stained 3D bioprinted cell-laden scaffolds at day 1 and 15. Viable cells are represented in green, and dead cells are shown in red. After 2 weeks of dynamic culture, some limited cell connections were observed in higher cell density groups (Figure 3D). Cell-laden scaffolds exhibited high cell viabilities after bioprinting (>90%) and after 2 weeks of daily mechanical loading (>85%) (Figure 3E). A slight decrease in viability was observed for lower cell density constructs after 2 weeks of loading. The mean cell density of osteocytes measured in the bone benchmark was 359 ± 74.6 cells/mm^2^ (Supplementary Table S1). Bioprinting with high cell densities produced scaffolds with similar day 1 cell densities (344.9 ± 139.7 cells/mm^2^) as the reference bone of the same donor (Supplementary Table S1). Consistent with previous results, a decrease in cell density was observed from day 1 to day 15 (Figure 3F).

### 3.2 Mineralization

**FIGURE 4:**
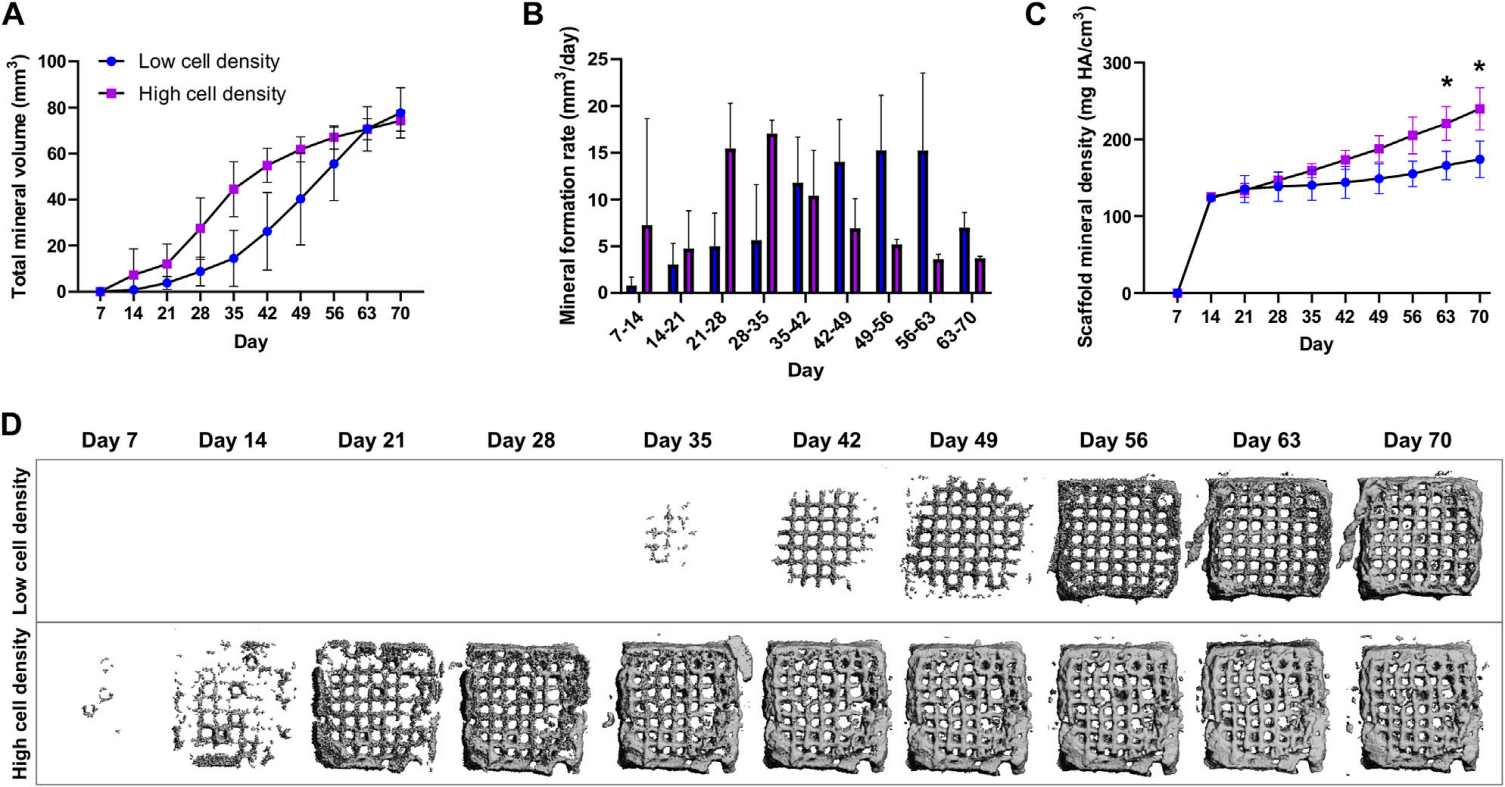
Time-lapsed micro-CT data of low and high cell density scaffolds with a mineral density above the threshold of 97.5 mg HA/cm^3^. **(A)** Total mineral volume, **(B)** Mineral formation rate (mineral volume changes over time) and, **(C)** Scaffold mineral density normalized to the first timepoint. **(D)** Time-lapsed 3D reconstructions of representative low and high cell density scaffolds. *p < 0.05, data are shown as mean ± standard deviation (*n* = 5).

Mineral formation and maturation were tracked by weekly micro-CT scans of cell-laden scaffolds. Both low and high cell density constructs were able to mineralize (Figure 4). However, while time-lapsed micro-CT images revealed similar endpoint mineral volumes, significant differences were found in the mineralization rates and mineral densities between the two cell density groups. Higher cell density constructs exhibited peak mineral formation rates in the earlier time points (28–35 days), while lower cell density constructs reached peak mineral formation after 49–56 days (Figure 4B). Notably, a significantly higher average mineral density of 230.8 ± 15 mg HA/cm^3^ was found in the higher cell density group compared to 176.9 ± 21.42 mg HA/cm^3^ in the low cell density group after 70 days of culture (Figure 4C).

### 3.3 Mechanics

**FIGURE 5:**
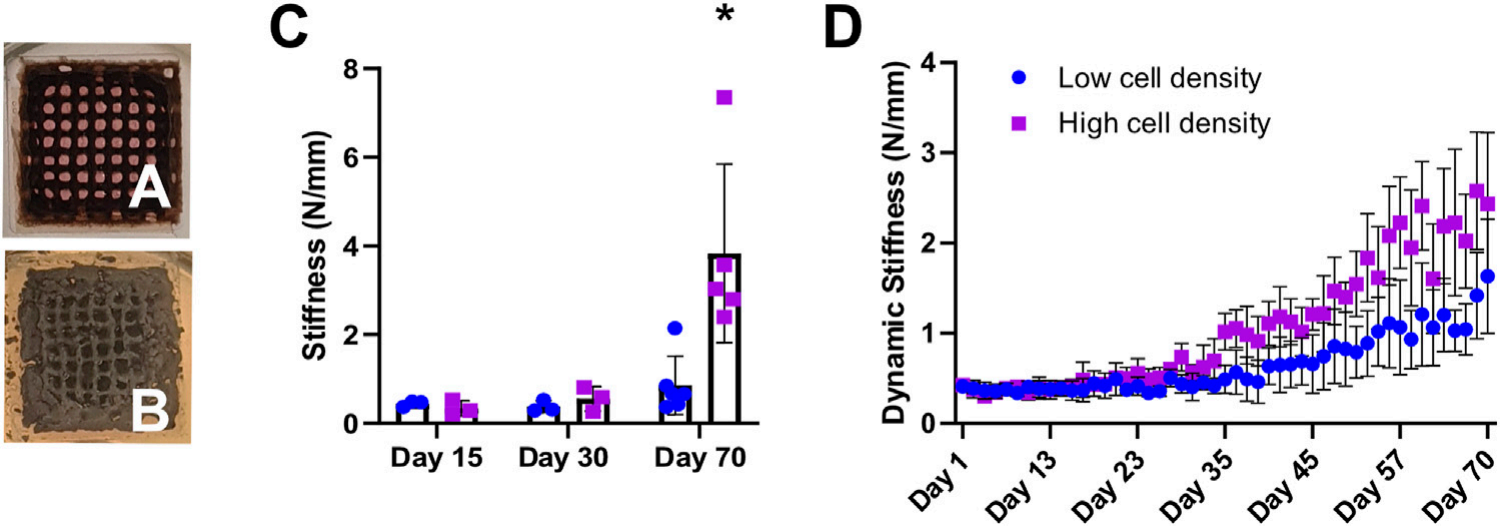
Representative photographs of 3D bioprinted scaffolds show constructs’ appearance during mineralization from day 1 **(A)** to day 70 **(B)**. **(C)** Destructive stiffness (N/mm) measured on days 15, 30 and 70. **(D)** Average dynamic stiffness measured from non-destructive daily loading data. *p < 0.05, data are shown as mean ± standard deviation (*n* = 5).

Mechanics were assessed using our in-house MSU in the form of (1) daily non-destructive measurements, referred to as dynamic stiffness and (2) unconfined uniaxial compression tests on days 15, 30 and 70. A 10-fold increase in stiffness was observed in high cell density scaffolds at day 70 compared to day 15 measurements, increasing from 0.34 ± 0.17 N/mm to 3.83 ± 2.01 N/mm at the endpoint (Figure 5C). Meanwhile, low cell density scaffolds only showed a 2-fold increase in stiffness during this time (0.45 ± 0.07 N/mm to 0.85 ± 0.65 N/mm) (Supplementary Table S2). Cell-laden scaffolds in both groups showed an increasing trend in dynamic stiffness throughout the study (Figure 5D). Scaffold maturation was observed in terms of increases in mineral volume, mineral density, stiffness as well as a visual change in appearance from a brown hydrogel template (Figure 5A) to a grey mineralized construct (Figure 5B).

### 3.4 Extracellular matrix characterization

**FIGURE 6:**
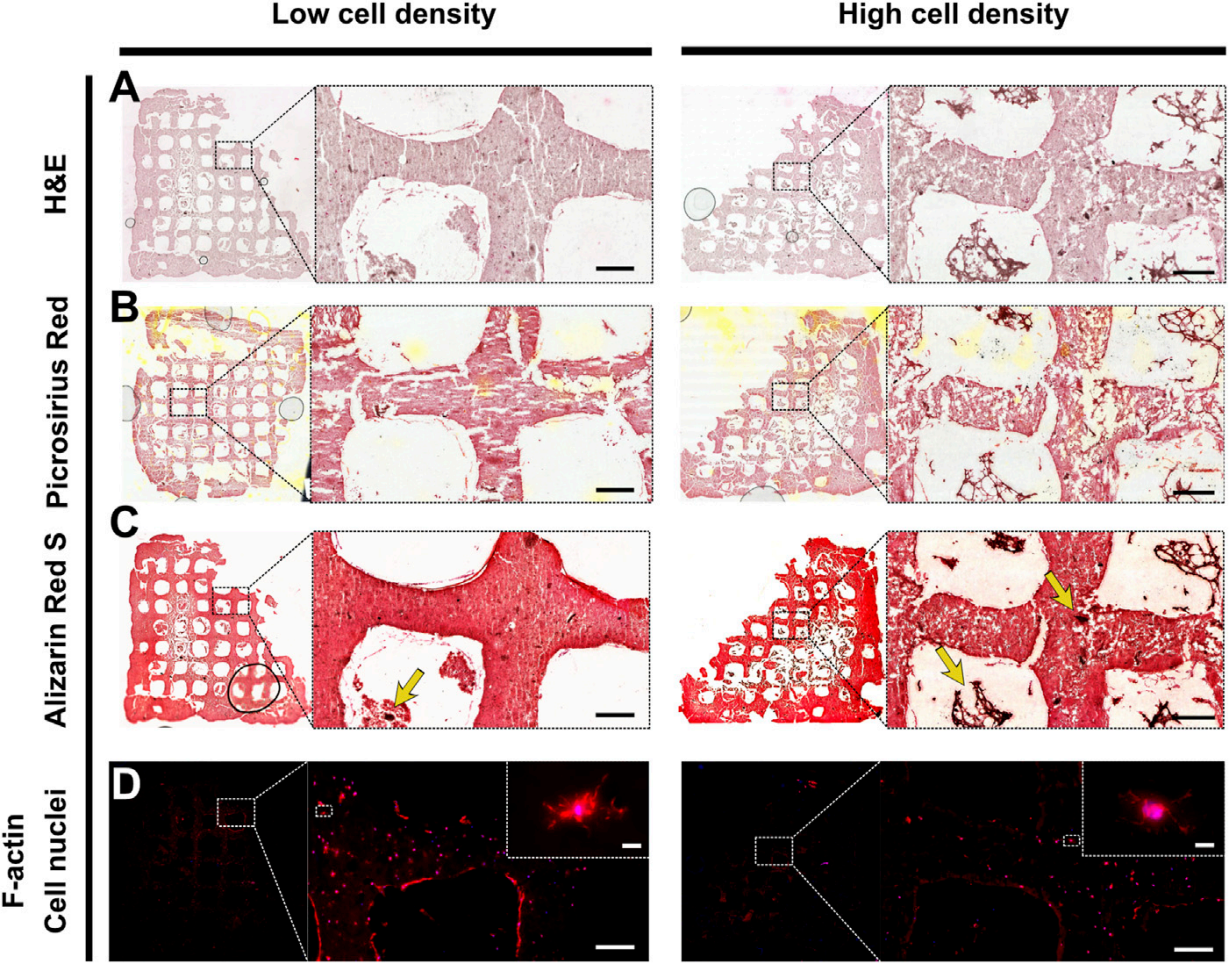
Representative histological and F-actin staining of low cell density scaffold (left) and high cell density scaffold (right). Brightfield imaging of **(A)** Hematoxylin and eosin (H&E), **(B)** Picrosirius red, **(C)** Alizarin red S staining. Yellow arrows indicate mineral nodules. Scale bar = 200 µm. **(D)** Fluorescence imaging of cell nuclei (blue) and F-actin (red). Scale bar single cell inlet = 10 µm.

After 70 days of culture, primary osteoblasts embedded in 3D-bioprinted alginate-gelatin-graphene oxide hydrogels were able to produce a mineralized extracellular matrix. Picrosirius Red Staining showed collagen (orange) presence, particularly in pericellular spaces and Alizarin Red S staining revealed mineral deposits throughout the constructs (Figure 6). In line with mineral density data, Alizarin Red S staining revealed more frequent intensely stained mineral nodules (Figure 6C) in high cell density scaffolds than low cell density scaffolds, indicating enhanced mineral maturation. Fluorescence imaging of F-actin and cell nuclei revealed in both groups the presence of embedded cells showing dendritic morphologies (Figure 6D).

### 3.5 Cell functionality

**FIGURE 7:**
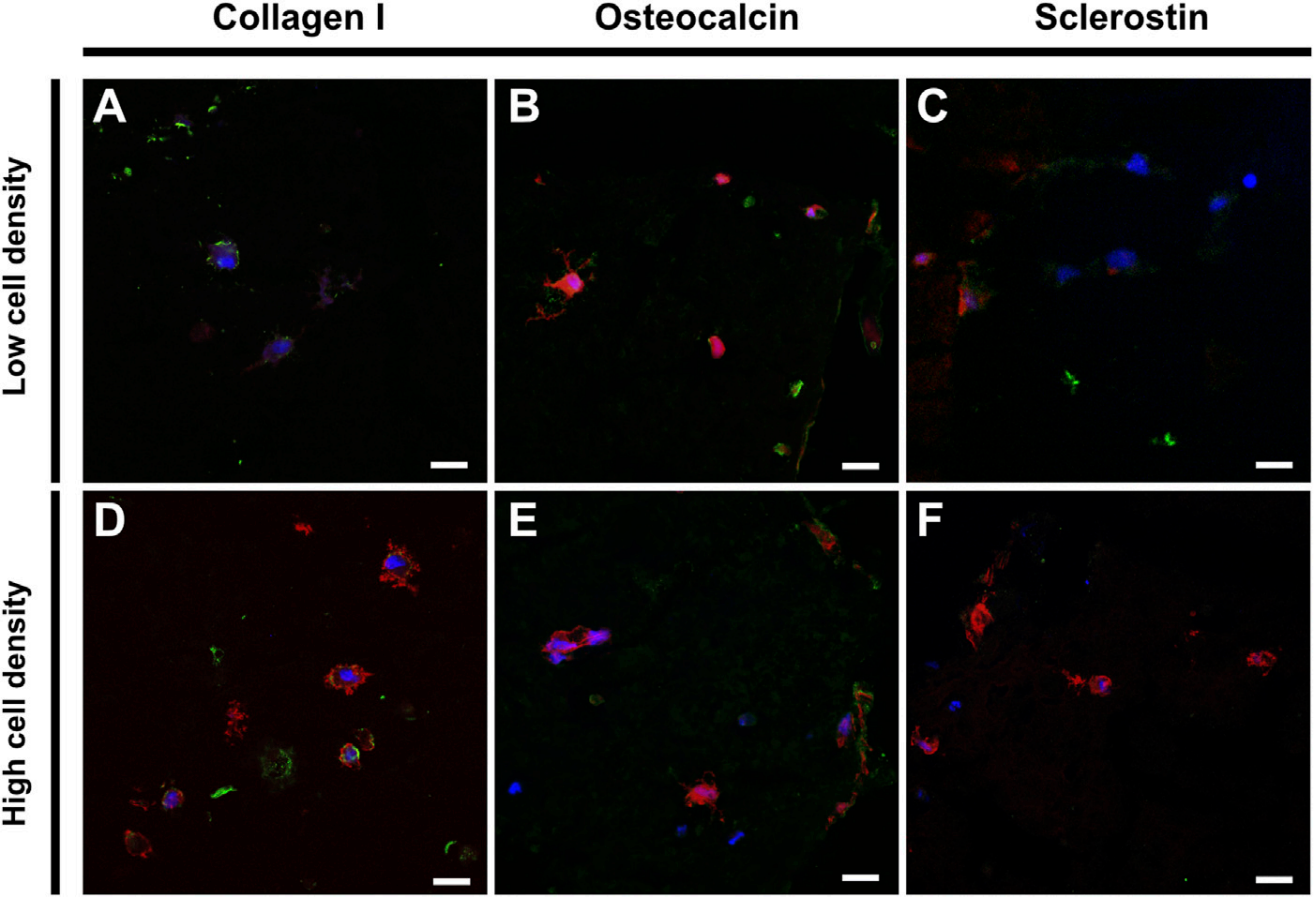
Immunohistochemical staining of low (top) and high (bottom) cell density scaffolds. Confocal imaging of cell nuclei (blue), F-actin (red) and **(A,D)** Collagen I (green), **(B,E)** Osteocalcin (green) or **(C,F)** Sclerostin (green). Scale bar = 20 µm.

Immunohistochemistry staining was performed to assess the functionality of embedded cells using osteogenic markers (Figure 7). Scaffolds of low and high cell density revealed comparable expression of collagen I and osteocalcin, where collagen I signal was mainly localized to the pericellular spaces as well as in the construct pores (Figure 6B and Figure 7). The mechanoregulated osteocyte marker sclerostin was used to assess the functionality and maturation of cells. Interestingly, lower sclerostin expression was observed in high cell density scaffolds (Figure 7F) (Supplementary Figure S5).

### 3.6 Cell morphology

**FIGURE 8:**
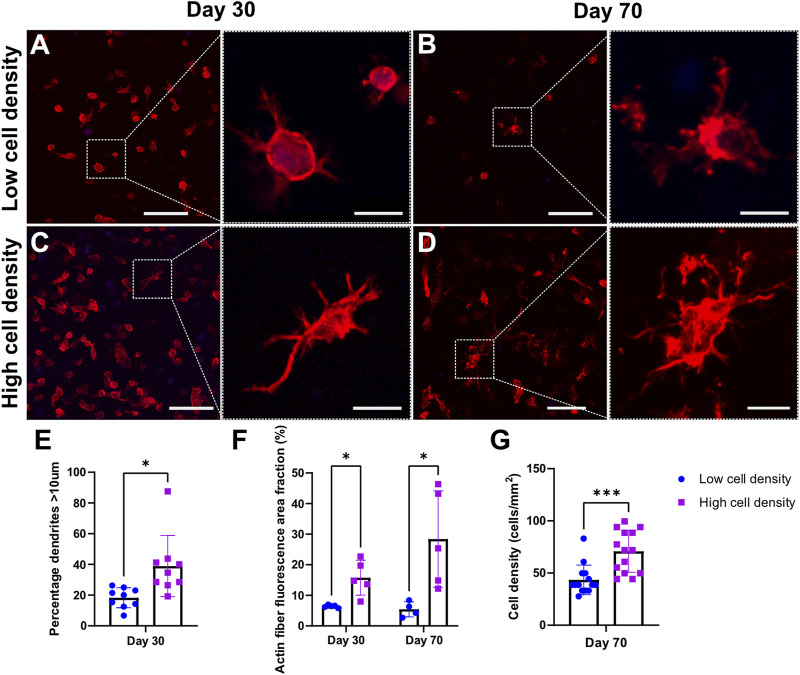
Cell morphology of F-actin (red) and cell nuclei (blue) staining. Confocal images of mechanically loaded scaffolds at day 30 **(A,C)** and day 70 **(B,D)**. Scale bar = 250 µm. High resolution insets showing cell morphology. Scale bar = 20 µm. Quantitative analysis of percentage of dendrites with length >10 μm at day 30 **(E)** and actin fiber fluorescence area fraction at days 30 and 70 **(F)**. Quantification of cell density of day 70 cryosections **(G)**. ***p < 0.001, data are shown as mean ± standard deviation (n = 3).

Within the constructs, we observed heterogenous cell morphologies in distinct regional distribution patterns indicating cellular self-organization and maturation (Supplementary Figures S2, S3). Cells embedded deep inside the hydrogel filaments resemble osteocyte-like cells or have a round morphology, while cells residing at the construct surface interface tend to adopt a flattened lining cell morphology similar to the cellular organization of bone (Figure 8D). In contrast to these encapsulated cells, we regularly found mobile osteoblast-like cells entering and spanning the construct pores (Figure 6A). Moreover, these cells demonstrated active production of extracellular matrix and subsequent mineralization (Figure 4 and Figure 6). Bioprinting at higher cell densities produced scaffolds with cells presenting a more dendritic morphology. In addition, various other cell morphologies could be observed in the scaffolds (Supplementary Figure S3). While dendrite formation was easily identified, limited interconnected networks were observed (Figures 8A–D). Actin fiber fluorescence area fraction and dendrite quantification revealed increased cell spreading morphology in high cell density scaffolds (Figures 8E, F). Quantification of cell density in high cell bioprinting density scaffolds remained at two-fold cell density after 70 days of dynamic culture as intended, suggesting this culture system can sustain cells during long-term dynamic culture (Figure 8G).

## 4 Discussion

### 4.1 Manufacturing pipeline

Bone organoids are 3D self-organized *in vitro* tissues built from osteoconductive biomaterials and stem cells or progenitor cells to create a biomimetic mineralized construct (Chen et al., 2022). In a preceding study, our group established a novel bone organoid technology that involves extrusion bioprinting of a novel bioink laden with hMSCs to create an open scaffold structure. This approach resulted in a uniform cell distribution throughout the structure rather than a surface cell gradient resulting from top seeding approaches (Zhang et al., 2022). Zhang et al., demonstrated that the uniform cell distribution within the elastic bioink mimics a physiological osteocyte network and facilitates mechanical stimulation in compression bioreactors by simulating *in vivo* loading characteristics. Here, we demonstrate that the established model can be adapted to a clinically available cell source. Our work aims to model human bone physiology, which entails a certain natural cell density, based on initial results from Zhang et al., and the cell density elucidated from our explants we chose to create two groups–the physiological cell density and the cell density that was previously deemed optimal (control group). For the present study, we successfully applied human donor-cells to this *in vitro* model of early bone formation (woven bone) and investigated the effect of two different cell bioprinting densities on cell-laden scaffold development over an extended period of 10 weeks. Our results indicate that human donor osteoblasts differentiate and mature timely and unimpededly in the microenvironment of our *in vitro* bone model, in line with hMSCs used previously (Zhang et al., 2022). Cell viability as well as scaffold maturation, mechanics and mineralization were significantly enhanced by matching initial cell density with patient-specific osteocyte density as assessed in bone samples prior to cell expansion.

### 4.2 Viability and cell printing density

During extrusion bioprinting, the bioink has a shear thinning effect allowing the material to flow out of the nozzle at cell-friendly pressure (Schwab et al., 2020). Most extrusion bioprinting protocols use cell densities of 1–10 × 10^6^ cells/mL of bioink depending on the cell source and bioink (Holzl et al., 2016; Zhang et al., 2021b). Printing at low cell densities reduces cell-cell interactions, while high cell densities alter the rheological properties of the bioink and may lead to cell death due to high shear stress experienced in the printing nozzle during extrusion (Holzl et al., 2016). High cell densities cause high cell to nozzle wall contact during extrusion, where cell membranes can be ruptured after being forced through a narrow aperture channel (Cidonio et al., 2019). Zhang et al. reported increased shear stress and reduced cell viability in alginate gelatin bioinks at a cell density of 15 × 10^6^ cell/mL (Zhang et al., 2020). Bone tissue engineering constructs are often reported in the range of 5–10 × 10^6^ cells/mL of bioink to ensure cell viability and function (Nicodemus and Bryant, 2008; Fedorovich et al., 2011; Cidonio et al., 2019; Zhang et al., 2020; Yang et al., 2022). The optimal cell density of *in vitro* bone constructs is debated; however, a minimal cell density is required to achieve extracellular matrix production, mineralization, and osteogenic marker expression (Ma et al., 2014). Zhou et al. reported that above a certain cell density, further increasing the cell density reduced cell function, osteogenic gene expression and mineralization (Zhou et al., 2011). We have shown more physiological cell bioprinting densities (10 × 10^6^ cells/mL) are associated with increased osteocyte-like cell development, mineral density, and stiffness with no adverse effects on cell viability. While even higher cell bioprinting densities may be more effective to increase mineralization, the scaffolds would be associated with reduced cell viability due to higher shear stresses (Zhang et al., 2020) during the printing process and the quality of the explanted bone may restrict the population of primary cells that can be derived within a reasonable number of passages. Furthermore, considerations such as reducing the scaffold volume to enable higher cell bioprinting densities are hindered by technical constraints, including mechanical loading device limitations and bioreactor design.

The intent of our study was to recapitulate the optimal cell bioprinting density previously reported for hMSC-laden scaffolds with an initial bioprinting density mimicking the physiological cell density of our donor. The initial bioprinting density was reported as number of cells per ml of bioink (cell/mL) as it is a parameter that can be easily controlled. However, as the cell density changes during *in vitro* culture, we could not report the groups as cells/mm³ limiting comparison with data from literature. Zhang et al. reported a decrease in mean cell density of scaffolds printed with 5 × 10^6^ cells/mL bioink from 380 ± 30.2 cells/mm^2^ after 7 days of culture to 227.8 ± 42 cells/mm^2^ after 21 days whereas scaffolds bioprinted with 15 × 10^6^ cells/mL bioink were reported to have a cell density of 412.7 ± 42 cells/mm^2^ after 21 days (Zhang et al., 2020). The cell density in our bone samples collected from surgery was measured to be 359 ± 74.6 cells/mm^2^. Based on this, we estimated the cell concentration in the bioink to be 10 × 10^6^ cells/mL to result in scaffolds with a comparable cell density to the donor bone benchmark. Printing with 10 × 10^6^ cells/mL bioink produced scaffolds with similar day 1 cell densities (344.9 ± 139.7 cells/mm^2^) as measured in the bone benchmark (Supplementary Table S1). After 70 days of culture, the cell density was observed to be 43.5 ± 14.2 cells/mm^2^ for the low cell density group and 70.8 ± 20.0 cells/mm^2^ for the high cell density group (Figure 8G). In healthy bone, the osteocyte density is reported to be 226.0 ± 26.75 cells/mm^2^ (Mahr et al., 2021). While the cell-laden scaffolds were designed to mimic the donor’s physiological cell density, cell density decreases over time below the level found in bone. As reported previously, this decrease may result from cell migration out of the scaffold into pores (Figure 6) and onto the bioreactor platform due to movement of cell culture medium inside the bioreactor (Seo et al., 2018; Zhang et al., 2020). Notably, this decrease was reported for static culture conditions. In the current dynamic loading environment, the motion of the bioreactor piston during mechanical loading physically deforms the hydrogel matrix and displaces cell culture medium creating fluid flow through the hydrogel which may exacerbate cell loss. This phenomenon of decreasing cell density over time should be considered and influences the ability of our constructs to accurately replicate the physiological cell density targeted in this study.

### 4.3 Mineralization

In order to non-destructively investigate mineral formation and maturation in 3D, time-lapse micro-CT scans were taken at weekly intervals. By increasing cell printing density, we were able to accelerate mineral maturation rates and reach higher mineral density (Figure 4). Proper thresholding to segment signal from background noise represents a crucial step in image analysis. A higher threshold than previously reported (Zhang et al., 2022) was selected in this work (97.5 mg HA/cm^3^ compared to 83.44 mg HA/cm^3^), to highlight differences particularly during the early stages of mineralization (Figure 4D), where mineral above the threshold appears at day 7 in higher cell density scaffolds, while lower cell density scaffolds require 4 weeks longer culture to reach similar mineral maturation levels (day 35). While it seems like the mineral volume in the low cell density group is still increasing at day 70, the total mineral volume in this model is defined by the volume of hydrogel extruded. The model does not induce appositional mineralization, which is also supported by the findings of Zhang et al. (Zhang et al., 2020; Zhang et al., 2022). Since the cells are embedded inside the hydrogel there is limited formation of extracellular matrix on the outside surfaces of the gel, rather newly synthesized proteins and minerals are incorporated into the hydrogel template. With higher initial cell bioprinting density, early mineral formation rate and mineral density were increased, however the overall mineralization pattern in the constructs remained unaltered (Supplementary Figure S6). While higher cell bioprinting densities show improved mineralization, for future clinical applications of the model, particularly in cases where limited material is available after surgery, lower bioprinting densities could be considered to allow for creation of a patient-derived model from limited starting cell numbers.

### 4.4 Mechanics

Mechanical analysis revealed a strong correlation between cell bioprinting density and stiffness (Figure 5). Moreover, scaffold mineral density and stiffness are well correlated, corroborating previous reports of mechanically loaded hMSC-laden scaffolds as well as natural bone (Zhang et al., 2022). Higher cell density scaffolds exhibited increased stiffness as compared to lower cell density scaffolds. The increase in cell density may improve cell-cell and cell-matrix interactions enabling faster mineralization, where an increase in mineral density subsequently results in stiffer constructs (Figure 4C and Figure 5D **)** (Daly et al., 2021; Zhang et al., 2022). Higher cell densities may enhance paracrine signaling and gap-junction communication in the construct, creating an environment supporting bone formation and more closely mimicking *in vivo* bone conditions (Brown et al., 1993; Lecanda et al., 1998).

### 4.5 Cell functionality, morphology, and extracellular matrix characterization

Our platform uses uniaxial compressive loading to apply physiological mechanical cues to enhance the 3D bioprinted cell-laden scaffold microenvironment. Several notable *in vitro* bone models have emerged using commercial hMSCs cultured under 3D dynamic conditions, yielding highly mineralized constructs, resembling osteocytes embedded in lacuna (Akiva et al., 2021; Zhang et al., 2022). Here, we have demonstrated osteoblasts derived from patients can also serve as viable cell sources to produce highly mineralized constructs, with osteoblastic cells (Supplementary Figure S3) and embedded cells resembling osteocyte-like features (Figures 7, 8), providing a more clinically relevant model.

In our analysis of bone biomarkers, we found expression of mid- (osteocalcin) and late- (sclerostin) osteogenic proteins as well as formation of dendritic cell processes and mineralization extracellular matrix, suggesting that the culture system supports maturation of primary cell-laden scaffolds towards functional *in vitro* bone models (Figure 7). Osteocalcin, expressed by late-stage osteoblasts, is involved in the regulation of osteoblast activity and serves as biomarker for mineral deposition and maturation *in vitro* and in clinical diagnostics (Neve et al., 2013). Both low and high cell density scaffolds expressed osteocalcin, supporting mineralization and maturation of the extracellular matrix. *De novo* collagen deposition was observed both in the pericellular space of the encapsulated cells as well as in the construct pores, acting as a three-dimensional support structure to facilitate cell differentiation and matrix mineralization (Figure 6B; Figure 7). Cells are known to migrate through and out of hydrogels (Salam et al., 2018; Kotlarz et al., 2023). Given that the primary osteoblasts were isolated based on their ability to migrate out of bone explants and adhere to tissue culture plastic, it is unsurprising that the cells retained their migratory phenotype and could be found in the macroscale pores between bioprinted filaments. As the embedded cells primarily adopt an osteocyte-like phenotype, they are not expected to produce large amounts of fibrillar collagen but remodel their pericellular space which is where most collagen in the constructs is found (Bonewald, 2011).

In our model, we found the presence of late-osteogenic maker sclerostin on a protein level, supporting the functionality of cells. While sclerostin staining results appear weaker than in previous reports using other antibodies (Akiva et al., 2021; Zhang et al., 2022), where staining was mainly localized adjacent to cells, the signal in cell-laden scaffolds appears similar to the positive antibody control of the donor’s bone (Supplementary Figure S4). While sclerostin expression was found in the low cell density scaffolds, the high cell density scaffolds expressed lower levels of sclerostin (Supplementary Figure S5). Previous *in vivo* and *in vitro* studies have shown mechanical loading decreases the expression of sclerostin (Robling et al., 2008; Webster et al., 2010; Moustafa et al., 2012; Spatz et al., 2015; Shu et al., 2017). Since our experimental loading protocol employs strain-controlled compression, the fixed amount of strain (1%) results in higher loads for stiffer constructs. Given that sclerostin is a mechanically regulated protein, we speculate this increased load leads to increased local strains and stresses and may contribute to a downregulation of sclerostin expression in the stiffer high cell density scaffolds. However, to establish a load-effect relationship of sclerostin in this model more research is required. An adaptive loading protocol could be employed in the future to explore this relationship further and more robust markers than sclerostin should be considered to confirm the presence of osteocytes in this model.

### 4.6 Limitations

The *in vitro* bone model presented in this study emulates mechanobiological cues to produce *de novo* mineralized tissue from donor specific bone cells. Due to the piloting character of our study to transition from hMSCs to primary human osteoblasts, it is limited to cells from a single human donor (Zhang et al., 2022). As cells were isolated from surgical waste bone based on tissue culture plastic-adherence without further characterization, there may be a non-homogenous population of cells derived from the donor. While our study presents a promising step towards personalization of the model, we acknowledge the need for further validation. In subsequent studies, additional cell-laden scaffolds will be fabricated from several pediatric human donors, from both sexes and different age groups both metabolically healthy and diseased. The absence of gene expression data in this study limit insights into cell differentiation state. Future studies should address these limitations to advance the *in vitro* bone model. The cell density data in this study was presented as cells/mm^2^ due to the techniques used to measure cell numbers, using a microscope to analyze sections, limiting its comparability to existing data in literature reported as cells/mm^3^. While aiming to reproduce a physiologically relevant cell density in the scaffolds, the number of cells decreased over the duration of the culture, falling below the density found in the donor’s bone. Our *in vitro* cultures are performed for 10 weeks, with a cell-laden hydrogel network as starting material, relying on *de novo* mineralized tissue formation (osteoid). In addition, there is no recruitment of new cells in the model or presence of osteoclasts. Thus, in comparison to real bone tissue with complex remodeled architecture, the mineral density and stiffness are not fully recapitulated. Furthermore, limited collagen formation was present in the model, despite the mechanical stimulation and presence of osteogenic differentiation factors. Future research should focus on developing an *in vitro* bone model with increased cellular complexity and implement multiomics analyses to gain a deeper understanding into the molecular mechanisms involved in early bone development in both healthy and pathologic human bone.

## 5 Conclusion

In this study, we established a pipeline for developing 3D bioprinted human bone-derived cell-laden scaffolds and investigated the effect of cell bioprinting density on mineral formation, stiffness, and cell morphology. Bioprinting with a higher, more physiological cell density significantly increased mineral density and stiffness across the 10-week culture period and demonstrated robust osteogenic protein expression. With baseline parameters and biomarkers established from healthy human donor osteoblasts, our forthcoming work will focus on working with diseased cells from pediatric patients with metabolic or genetic bone disorders. Ultimately, we aspire to creating a clinically validated *in vitro* bone model for studying individual pathomechanisms and phenotypic heterogeneity, providing reliable biomarkers that facilitate clinical decision making (e.g., predicting disease trajectories) and enabling advanced drug development and testing in the emerging field of precision medicine.

## Data Availability

The raw data supporting the conclusion of this article will be made available by the authors, without undue reservation.
