# Supplementary material for: Physiological cell bioprinting density in human bone-derived cell-laden scaffolds enhances matrix mineralization rate and stiffness under dynamic loading
Source: Front Bioeng Biotechnol. 2024 Feb 14;12:1310289. doi: 10.3389/fbioe.2024.1310289 (PMC10900528; doi:10.3389/fbioe.2024.1310289)
Supplement: Supplementary file 1 [file DataSheet1.PDF]

Physiological cell bioprinting density in human bone-derived cell-laden scaffolds enhances matrix mineralization rate and stiffness under dynamic loading

Supporting Information

*Anke de Leeuw[1], Reto Graf[1], Pei Jin Lim[2], Jianhua Zhang[1], Gian Nutal Schädli[1], Sheila Peterhans[1], Marianne Rohrbach[2], Cecilia Giunta[2], Matthias Rüger[1,3], Marina Rubert[1], Ralph Müller[1]**

1. Institute for Biomechanics, ETH Zurich, Gloriastrasse 37/39, 8092 Zurich, Switzerland
2. Connective Tissue Unit, Division of Metabolism and Children's Research Center, University Children's Hospital Zurich, University of Zurich, Zurich, Switzerland
3. Department of Pediatric Orthopaedics and Traumatology, University Children's Hospital Zurich, Zurich, Switzerland

*Email: ram@ethz.ch

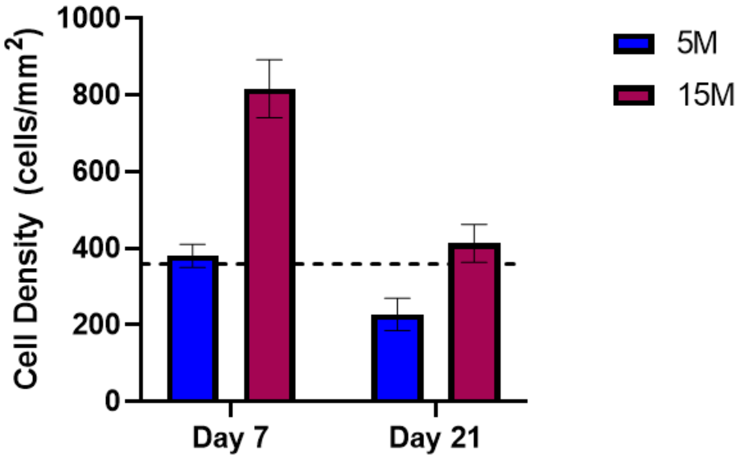

Supplementary Figure 1. Cell density of hMSC-laden scaffolds from previous studies by Zhang et al. [1] printed with $5x10^6$ cells/ml of bioink (5M) and $15x10^6$ cells/ml (15M) compared to osteocyte cell density of donor bone (dotted line).

[1] J. Zhang, E. Wehrle, P. Adamek, G.R. Paul, X.H. Qin, M. Rubert, R. Muller, Optimization of mechanical stiffness and cell density of 3D bioprinted cell-laden scaffolds improves extracellular matrix mineralization and cellular organization for bone tissue engineering, Acta Biomater 114 (2020) 307-322.

	LOW CELL DENSITY	HIGH CELL DENSITY
**DAY 1**	252.6 ± 57.0 cells/mm^2	344.9 ± 139.7 cells/mm^2
**DAY 15**	108.2 ± 26.3 cells/mm^2	171.3 ± 48.9 cells/mm^2
**DAY 70**	43.5 ± 14.2 cells/mm^2	70.8 ± 20.0 cells/mm^2
**BONE BENCHMARK**	359 ± 74.6 cells/mm^2	

Supplementary Table 1. Cell density (cells/mm^2) after bioprinting (day 1) and after two (day 15) and 10 weeks (day 70) of daily mechanical loading.

	LOW CELL DENSITY	HIGH CELL DENSITY
**DAY 15**	0.45 ± 0.07 N/mm	0.34 ± 0.17 N/mm
**DAY 30**	0.38 ± 0.13 N/mm	0.55 ± 0.28 N/mm
**DAY 70**	0.85 ± 0.65 N/mm	3.83 ± 2.01 N/mm

Supplementary Table 2. Destructive stiffness measurements (N/mm) after 15, 30 and 70 days of mechanical loading.

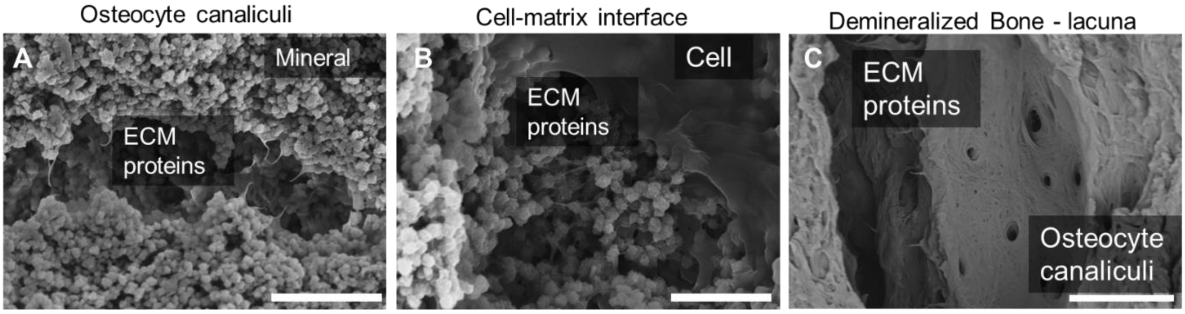

Supplementary Figure 2. Scanning electron microscopy (SEM) images of (A) osteocyte canaliculi with ECM protein filaments in 3D bioprinted cell-laden scaffold cryosection, (B) cell-matrix interface with ECM proteins, mineral nodules, and embedded primary cell. (C) Osteocyte canaliculi with extracellular matrix (ECM) protein filaments demineralized bone benchmark.

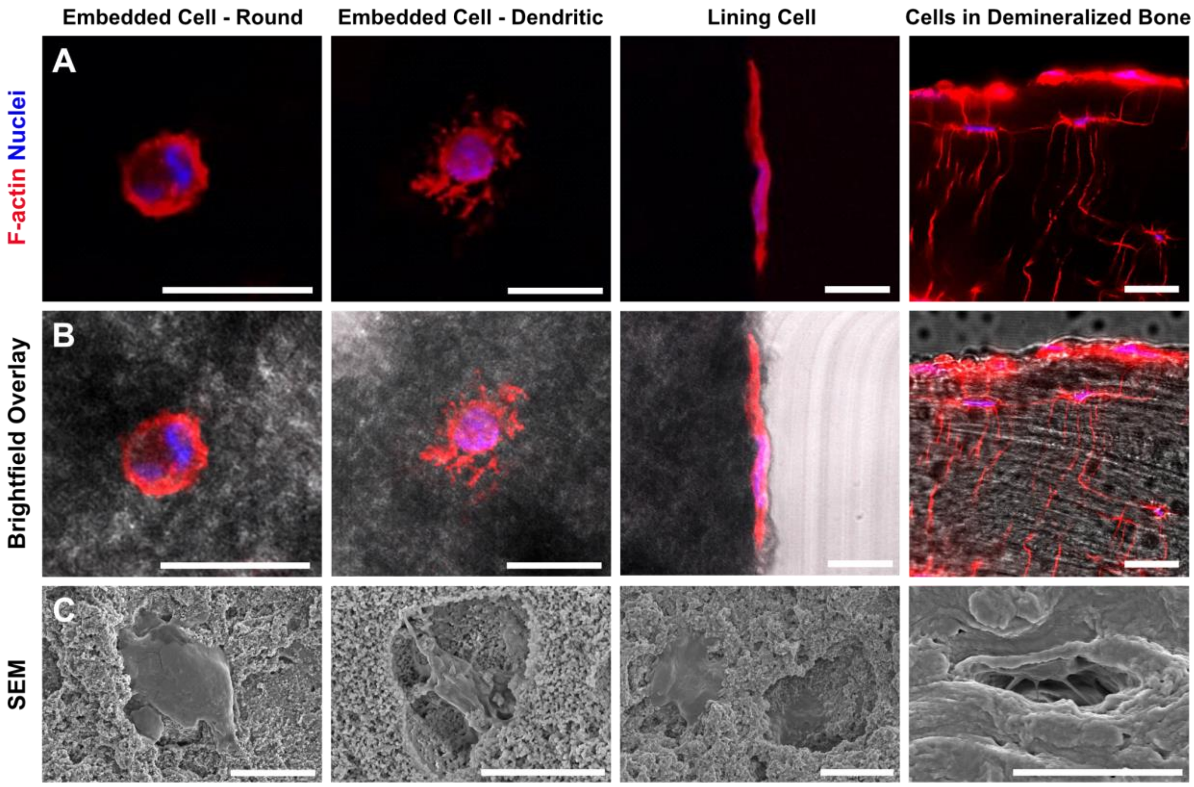

Supplementary Figure 3. (A) Confocal images of F-actin (red) and cell nuclei (blue) representing distinct cell phenotypes in different regions of the construct (embedded inside hydrogel filament or lining scaffold pore) compared to osteoblast and osteocyte morphology in demineralized bone benchmark with (B) brightfield overlay. Scale bar = 20 µm. (C) Scanning electron microscopy (SEM) images of local cellular microenvironment. Scale bar = 10 µm.

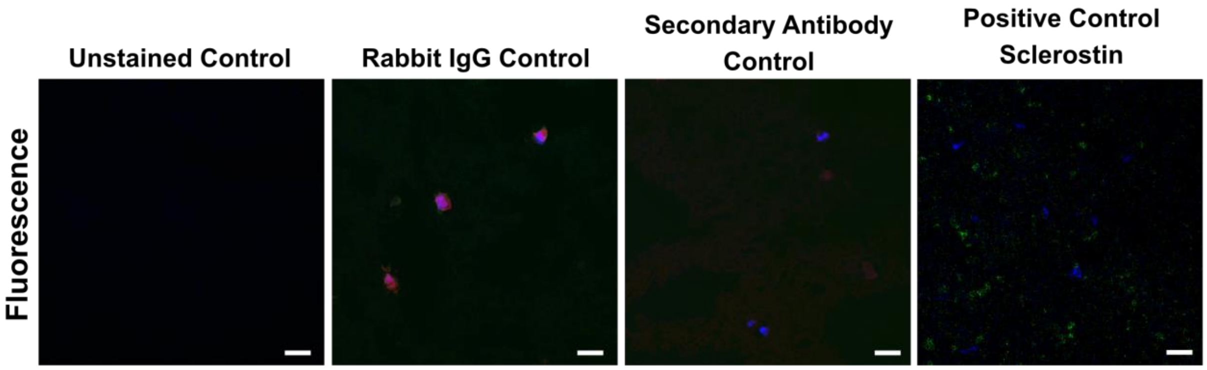

Supplementary Figure 4. Assessment of the immunostaining specificity of antibodies in low and high cell density scaffold cryosections. Confocal images of F-actin (red), cell nuclei (blue), rabbit IgG control (green). Positive control demineralized bone cryosection showing cell nuclei (blue), sclerostin (green). Scale bar = 20 µm.

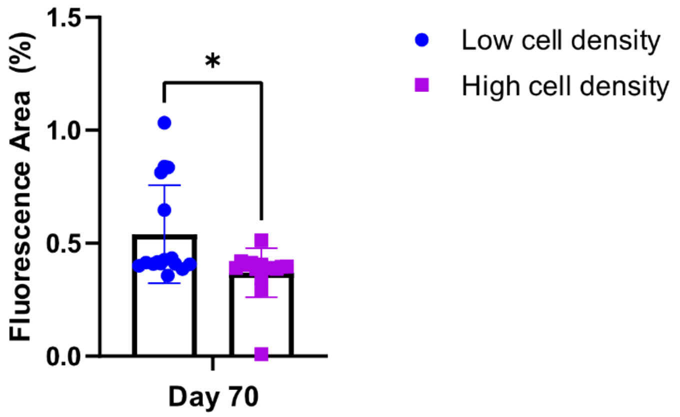

Supplementary Figure 5. Assessment of fluorescence area (%) in anti-sclerostin stained scaffold cryosections. A Student's t-test revealed significantly higher fluorescence area in ($p = 0.014$) in the low cell density group.

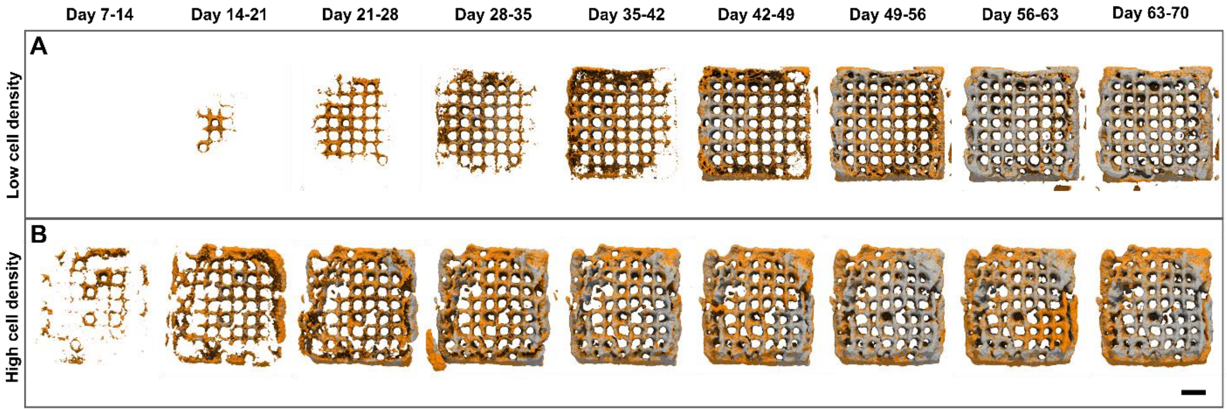

Supplementary Figure 6. Representative images of 3D bioprinted cell-laden scaffolds of (A) low cell density and (B) high cell density. Visualization of mineral formation (orange) and quiescent areas (grey) in 3D registered timelapsed micro-CT images. Scale bar = 2 mm.

Methods: To visualize the location of newly formed mineral volumes, two consecutive scans were registered with each other within a weekly interval [2,3]. Briefly, the images were gaussian filtered (σ = 1.2, support = 1), binarized with a global threshold corresponding to a mineral density of 97.5 mg HA/cm^3 and then the largest connected component was kept (Python 3.11.6, Scipy 1.10.1, Scikit-Image 0.21.0). The binarized images were registered using a rigid least-squared registration method to calculate rotation and translation registration parameters. These registration parameters were applied to the raw greyscale images and basic image processing steps were repeated to obtain the binarized image. Voxels that were present only in the follow-up scan are displayed in orange, and those present in both time points in grey. Due to the mechanical loading some deformation and translation of the mineralized volumes occurred, therefore voxels that were only present in the first time-point were ignored. Consequently, some of the volumes may have been labelled orange because of translation and deformation.

[2] LAMBERS, F. M., KOCH, K., KUHN, G., RUFFONI, D., WEIGT, C., SCHULTE, F. A. & MULLER, R. 2013. Trabecular bone adapts to long-term cyclic loading by increasing stiffness and normalization of dynamic morphometric rates. *Bone,* 55**,** 325-34.

[3] LAMBERS, F. M., SCHULTE, F. A., KUHN, G., WEBSTER, D. J. & MÜLLER, R. 2011. Mouse tail vertebrae adapt to cyclic mechanical loading by increasing bone formation rate and decreasing bone resorption rate as shown by time-lapsed in vivo imaging of dynamic bone morphometry. *Bone,* 49.
